# Myeloablative conditioning with thiotepa-busulfan-fludarabine does not improve the outcome of patients transplanted with active leukemia: final results of the GITMO prospective trial GANDALF-01

**DOI:** 10.1038/s41409-022-01626-5

**Published:** 2022-04-12

**Authors:** Francesca Bonifazi, Chiara Pavoni, Jacopo Peccatori, Fabio Giglio, Mario Arpinati, Alessandro Busca, Paolo Bernasconi, Anna Grassi, Anna Paola Iori, Francesca Patriarca, Lucia Brunello, Carmen Di Grazia, Angelo Michele Carella, Daniela Cilloni, Alessandra Picardi, Anna Proia, Stella Santarone, Roberto Sorasio, Paola Carluccio, Patrizia Chiusolo, Alessandra Cupri, Mario Luppi, Chiara Nozzoli, Donatella Baronciani, Marco Casini, Giovanni Grillo, Maurizio Musso, Francesco Onida, Giulia Palazzo, Matteo Parma, Stefania Tringali, Adriana Vacca, Daniele Vallisa, Nicoletta Sacchi, Elena Oldani, Arianna Masciulli, Angela Gheorghiu, Corrado Girmenia, Massimo Martino, Benedetto Bruno, Alessandro Rambaldi, Fabio Ciceri, on behalf of GITMO

**Affiliations:** 1 IRCCS Azienda Ospedaliero-Universitaria di Bologna, Bologna, Italy; 2 Department of Oncology and Hematology Azienda Socio-Sanitaria Territoriale Papa Giovanni XXIII, Bergamo, Italy; 3 Department of Onco-Hematology - Hematology and Bone Marrow Transplantation, IRCCS San Raffaele Scientific Institute, Milan, Italy; 4 Vita-Salute San Raffaele University, Milan, Italy; 5 SSD Trapianto Cellule Staminali, AOU Città della salute e della Scienza, Torino, Italy; 6 Centro trapianti di cellule staminali ematopoietiche, UOC Ematologia, Fondazione IRCCS Policlinico San Matteo, Pavia, Italy; 7 Ematologia, AOU Policlinico Umberto 1, Sapienza Università di Roma, Roma, Italy; 8 Clinica Ematologica e Centro Trapianti, ASUFC, Udine; DAME, Università di Udine, Udine, Italy; 9 di Ematologia, Azienda Ospedaliera SS Antonio e Biagio e Cesare Arrigo, Alessandria, Italy; 10 Ematologia e Centro Trapianti. IRCCS Ospedale Policlinico San Martino, Genova, Italy; 11 SSD UTIE e Terapie Cellulari, Dipartimento Scienze Mediche, Fondazione Casa Sollievo della Sofferenza, San Giovanni Rotondo (FG), Italy; 12 Department of Clinical and Biological Sciences, University of Turin, Turin, Italy; 13 Rome Transplant Network, Department of Biotecnology and Prevention, Tor Vergata University, Rome, Italy; 14 Stem Cell Transplant Program of AORN Cardarelli, Naples, Italy; 15 Ematologia e Trapianto CSE, AO San Camillo-Forlanini, Roma, Italy; 16 Terapia Intensiva Ematologica- Ospedale Civile, Pescara, Italy; 17 S.C. Ematologia, A.O. S. Croce e Carle, Cuneo, Italy; 18 Department of Emergency and Organ Transplantation (D.E.T.O.), Hematology Section, University of Bari, Bari, Italy; 19 Dipartimento di Diagnostica per Immagini, Radioterapia Oncologica ed Ematologia, Fondazione Policlinico Universitario A. Gemelli IRCCS, Roma, Italy; 20 Sezione di Ematologia, Dipartimento di Scienze Radiologiche ed Ematologiche, Università Cattolica del Sacro Cuore, Roma, Italy; 21 Unità di Trapianto di Midollo, Divisione di Ematologia, Azienda Ospedaliera Policlinico di Catania, Catania, Italy; 22 Department of Medical and Surgical Sciences Unimore Azienda Ospedaliera Universitaria di Modena, Modena, Italy; 23 Department of Cellular Therapies and Transfusion Medicine, Careggi Hospital, Florence, Italy; 24 Ematologia e TMO Ospedale Oncologico “A. Businco”, Cagliari, Italy; 25 Ematologia e TMO, Bolzano, Italy; 26 GOM Niguarda Milano, Milano, Italy; 27 UOC di Oncoematologia e TMO Dipartimento Oncologico “La Maddalena” Palermo, Palermo, Italy; 28 IRCCS Ca’ Granda Ospedale Maggiore Policlinico Centro Trapianti Midollo Osseo - UOC Ematologia - Università degli Studi di Milano Dipartimento di Oncologia e Emato-Oncologia, Milano, Italy; 29 Ematologia - Taranto, Taranto, Italy; 30 Divisione di Ematologia e Centro Trapianti di Midollo, Ospedale San Gerardo, Monza, Italy; 31 AOR Villa Sofia Cervello, Dipartimento Oncologia, UOSD Unità Trapianti di Midollo Osseo, Palermo, Italy; 32 UO Centro Trapianti di Midollo Osseo Presidio Ospedaliero R. Binaghi, Cagliari, Italy; 33 UO Ematologia e CTMO, Piacenza, Italy; 34 IBMDR - E.O. Ospedali Galliera, Genova, Italy; 35 FROM-Fondazione per la Ricerca Ospedale di Bergamo, Bergamo, Italy; 36 Trials Office GITMO Gruppo Italiano per il Trapianto di Midollo Osseo, cellule staminali emopoietiche e terapia Cellulare, Genova, Italy; 37 Stem Cell Transplant and Cellular Therapies Unit, Grande Ospedale Metropolitano “BMM”, Reggio Calabria, Italy; 38 Dipartimento di Biotecnologie Molecolari e Scienze per la Salute - Università di Torino, Torino, Italy; 39 University of Milan, Milan, Italy

**Keywords:** Medical research, Haematological diseases

## Abstract

The outcome of refractory/relapsed (R/R) acute leukemias is still dismal and their treatment represents an unmet clinical need. However, allogeneic transplantation (allo-HSCT) remains the only potentially curative approach in this setting. A prospective study (GANDALF-01, NCT01814488; EUDRACT:2012-004008-37) on transplantation with alternative donors had been run by GITMO using a homogeneous myeloablative conditioning regimen with busulfan, thiotepa and fludarabine while GVHD prophylaxis was stratified by donor type. The study enrolled 101 patients; 90 found an alternative donor and 87 ultimately underwent allo-HSCT. Two-year overall survival of the entire and of the transplant population (primary endpoint) were 19% and 22%, without significant differences according to disease, donor type and disease history (relapsed vs refractory patients). Two-year progression-free survival was 19% and 17% respectively. The cumulative incidences of relapse and non-relapse mortality were 49% and 33% at two years. Acute grade II-IV and chronic GVHD occurred in 23 and 10 patients. Dose intensification with a myeloablative two-alkylating regimen as sole strategy for transplanting R/R acute leukemia does seem neither to improve the outcome nor to control disease relapse. A pre-planned relapse prevention should be included in the transplant strategy in this patient population.

## Introduction

A proportion of patients (10–40%) with acute myeloid leukemia (AML) fails to achieve a complete remission (CR) after two courses of intensive chemotherapy (IC) and 50-70% of those achieving CR will eventually relapse [1]. While their management is still controversial, the prognosis is quite evenly dismal, independently of the treatment approach, including the option of allogeneic hematopoietic stem cell transplantation (allo-HSCT), which however maintains a beneficial role [2, 3]. Similarly, the fate of patients with refractory or relapsed (R/R) acute lymphoblastic leukemia (ALL) disease still have a poor prognosis in term of overall survival [4] although the approved immunotherapeutic agents lead to very high rate of remission, even at the molecular level [5, 6] Allo-HSCT performs best in first complete remission (CR1) while in case of active disease shows all the limitations mainly due to the high cumulative incidence of relapse CIR) and non-relapse mortality (NRM) [3, 7].

However, different clinical outcomes have been reported in this setting, largely due to the heterogeneity of criteria adopted to define refractoriness and diverseness of treatment. The old ELN criteria [8] defined resistant leukemia after induction as persistence of blasts in patients alive after 7 days from treatment start or later while several studies defined refractory disease as persistence of leukemic blasts after two courses of intensive chemotherapy [2, 9].

Diverseness of treatment includes at least the heterogeneity of conditioning regimen and the choice of the transplant time strategy (upfront transplant versus attempts to induce a pre-transplant CR).

For all these reasons, in 2013 a prospective multicenter study, named GANDALF-01 (**G**ITMO **A**gainst **N**on-responding an**D A**cute Leukemia **F**ailures for transplant R/R leukemias), including an homogeneous myeloablative dual alkylator-conditioning regimen in patients lacking an HLA identical sibling donor was launched. Due to the poor prognosis of allo-HSCT with active disease in acute leukemias the Italian Transplant Programs were not allowed to proceed to an allotransplant from an unrelated donor (URD) in this setting of patients until the study run, which definitively changed that policy. The antileukemic strategy of the study was based on the need of a homogeneous truly myeloblative conditioning (MAC), being easily adopted by all the Italian centers. The choice of a uniform preparative regimen was justified by the need of limiting the diverseness of treatment within a prospective trial, even across different diseases (AML and ALL). The combination of thiotepa, busulfan, and fludarabine (TBF), initially designed for cord blood transplantation of patients with active disease [10, 11] and then widely used in Italy in the setting of haploidentical transplants [12–15], foresees the combination of two drugs, busulfan and thiotepa, with an optimal penetration into central nervous system, mimicking the radiation therapy. The exclusion of TBI, which is associated with lower relapse, at least in young adult ALL [16, 17] but not always to an increased survival in other settings [18, 19] was due to the limited availability of TBI in a population of patients needing an urgent transplant.

## Materials and methods

### Study design and inclusion criteria

A phase II multicenter (supplementary table 1) open-label study on allo-HSCT from URD, CB and family haploidentical donors in patients with active acute leukemia was promoted by GITMO in 2013 with the objective of increasing OS. The primary endpoint was the OS at 2 years of all patients enrolled into the study (either transplanted or not); secondary endpoints were the cumulative incidence of NRM, leukemia relapse (CIR), acute and chronic GVHD, primary engraftment, progression-free survival (PFS). Before the study run in Italy it was allowed to activate an URD search but not to proceed to transplant for patients suffering from acute leukemia with active disease. Accordingly, the patients activating an URD search at diagnosis and then becoming resistant/refractory could not proceed to transplant with active disease as well as those patients for whom an unrelated search was not yet activated early and then became either resistant or refractory. With the approval of the study instead both the former and the latter patients could finally proceed to potentially benefit from transplant option.

Inclusion criteria were adult patients (18–70 years) diagnosed of acute leukemia, with either a chemo-resistant relapse (relapsed patients) or primary induction failure (refractory patients), for whom an URD search (adult and cord blood search) at the Italian Bone Marrow Donor Registry (IBMDR) was activated, with Performance Status ECOG ≤ 3 and a life expectancy not severely limited by concomitant illness. The definition of refractoriness was defined as a failure to achieve a CR after at least one cycle of intensive chemotherapy.

Exclusion criteria were previous allo-HSCT, availability of a matched related donor and any active uncontrolled infection.

The study foresaw a homogenous approach for conditioning regimen while the GVHD prevention strategy was stratified according to donor type. The final choice of the donor was left to the center policy. Anti-infectious strategy was left to the center guidelines and a sub-study with micafungin was proposed to the participating centers as primary fungal prophylaxis (not reported here).

The study was conducted according to the Helsinki declaration, after approval of ethical committees of each participating center. All patients signed a written consent. The study was registered at NCT as NCT01814488 and EUDRACT as 2012-004008-37.

### Transplant procedures

All patients enrolled were patients for whom an URD search at the IBMDR was activated at any time, either at diagnosis of acute leukemia or after diagnosis of refractoriness/chemo-resistant relapse. For the patients activating the URD search at diagnosis and then becoming resistant/refractory, the study entry was immediately possible while for those ones not activating the URD search at diagnosis for any reasons and therefore after becoming resistant/refractory, the study entry was possible only after HLA typing and unrelated search activation.

The criteria for unrelated, CB and haploidentical donor search are reported on supplementary Table 2. A MAC regimen including thiotepa (10 mg/kg, total dose), busulfan (9.6 mg/kg), and fludarabine (150 mg/mq) (TBF) was given to all patients, while GVHD prophylaxis was stratified according to donor type: either anti-thymocyte globulin (ATG, 6 mg/kg) or anti-T lymphocyte globulin (ATLG, 30 mg/kg) was added to cyclosporin with either short-term methotrexate (URD) or mycophenolate mofetil (CB transplant); GVHD was instead prevented by post-transplant cyclophosphamide (PT-Cy), tacrolimus and mycophenolate mofetil [20] or ATLG (20 mg/kg), cyclosporin, short-term methotrexate, mycophenolate mofetil, and basiliximab [12] in haploidentical transplants, according to the center policy.

### Sample size calculation

Criteria for sample size assessment did not refer to a formal statistical power calculation because of a lack of detailed and focused data into this setting and the absence of alternative curative options. Therefore, by means of this study, GITMO promoted, for the first time in Italy, a study allowing patients with active leukemia to proceed to an allo-HSCT with the aim to collect outcome variables in the widest and most representative cohort of this specific patient population, independently of the type of donor (haploidentical, URD, CB) finally selected. The choice of 80 patients transplanted was based on feasibility reasons following the GITMO estimation on Italian transplant activity pointing to an estimated accrual of 40 patients per year over a 24-month enrollment time.

### Statistical analysis

All patients enrolled in the study were included in the intention to transplant (ITT) analysis. This population was called overall population. Specific analyses were done only on the transplant cohort.

Survival outcomes included in the analysis were OS, PFS, NRM, and cumulative incidence of relapse (CIR).

In the overall population, OS and PFS were calculated using as starting times both the time of study recruitment and the time of diagnosis of resistance/refractoriness, which guided the ITT (in the supplementary material as Supplementary Figs. 1 and 2)

In the transplant cohort instead OS was calculated from transplant to death. PFS, NRM, and CIR were calculated from transplant to relapse or death, whichever occurred first.

OS was estimated using the Kaplan–Meier method and the log-rank test was applied to test differences between groups. Follow-up was updated on April 2021. NRM and CIR were estimated using cumulative incidence function, considering relapse and death as a competing event, respectively, and the Gray’s non-parametric test was used to assess group differences. Univariate analysis was performed by fitting Cox models for OS and Fine and Gray models for NRM and CIR. Hazard ratio with 95% confidence intervals were reported. Transplant in the overall population and acute and chronic GvHD in transplant cohort were all considered as time-dependent factors and their unadjusted effect on survival outcomes was tested performing Cox models with time-dependent covariates. Transplant effect was graphically illustrated by Simon–Makuch plot. All reported *p* values were two-sided. All the analyses were performed with R software (version 4.0).

## Results

**Fig. 1:**
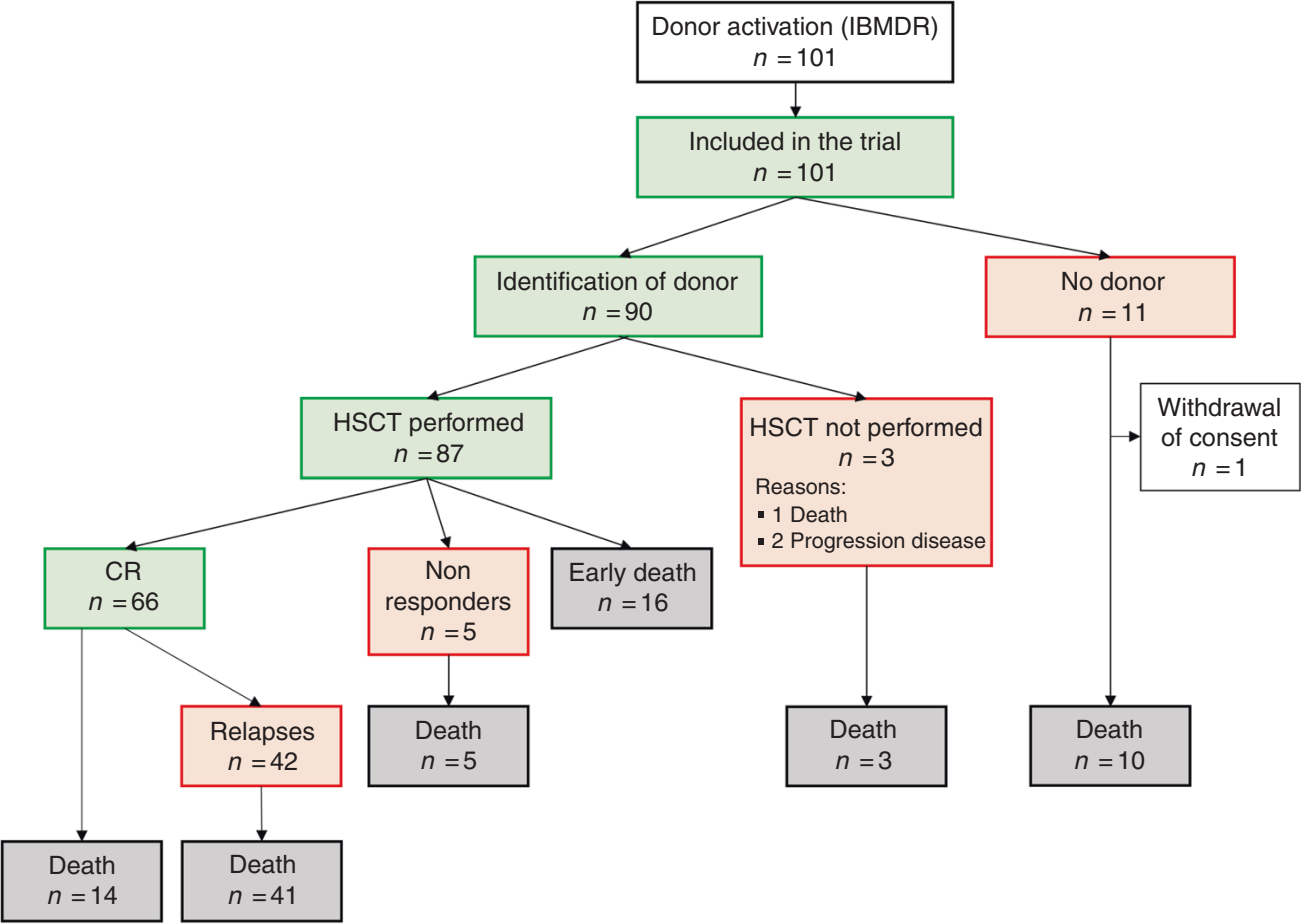
Consort diagram. IBMDR Italian Bone Marrow Donor Registry, HSCT allogeneic hematopoietic stem cell transplantation, CR complete remission.

One hundred and one patients (Fig. 1) activated the URD search at the IBMDR and were enrolled (supplementary table 1). For 90 of them a suitable donor was identified; 87 of the 90 ultimately underwent allo-HSCT with a median time interval from donor search activation to transplant of 106 days (interquartile range: 56–155 days). The median time from diagnosis of refractoriness/resistant relapse to transplant was 91 days (interquartile range: 49–156) while the median time from enrollment to transplant was only 18 days (interquartile range: 9–43); this short delay is justified by the new chance offered by GANDALF trial to proceed to transplant in patients with a donor already found and waiting for the approval of the study to proceed to transplant, that could not be performed before the study approval. In the transplant population, 60.3% of the patients had received ≥2 courses of intensive chemotherapy. Detailed clinical characteristics of the study population were reported in Table 1.

**Table 1:** Characteristics of the patients.

	All patients enrolled (*n* = 101)	Transplanted patients (*n* = 87)	Relapsed patients (*n* = 40)	Refractory patients (*n* = 47)	Non transplanted patients (*n* = 14)
Age (years), median (range)	54 (19–69)	52 (19–69)	46 (19–67)	54 (23–69)	62.5 (20–68)
Sex					
Female	45 (44.6)	40 (46)	20 (50)	20 (42.6)	5 (35.7)
Male	56 (55.4)	47 (54)	20 (50)	27 (57.4)	9 (64.3)
Previous auto HSCT	9 (8.9)	8 (9.2)	5 (12.5)	3 (6.4)	1 (7.1)
Diagnosis					
Acute myeloid leukemia	93 (92.1)	82 (94.0)	36 (90.0)	46 (97.9)	11 (78.6)
de novo	79 (84.9)	69 (84.1)	34 (94.4)	35 (76.1)	10 (90.9)
Secondary	13 (14.0)	12 (14.6)	2 (5.6)	10 (21.7)	1 (9.1)
Therapy-related	1 (1.1)	1 (1.2)	0	1 (2.2)	0
Acute Lymphoblastic Leukemia	8 (7.9)	5 (5.7)	4 (10.0)	1 (2.1)	3 (21.4)
de novo	7 (87.5)	4 (80.0)	3 (75)	1 (100)	3 (100)
Secondary	1 (12.5)	1 (20.0)	1 (25)	0	0 (0)
B-lineage	7 (87.5)	4 (80.0)	3 (75)	1 (100)	3 (100)
T- lineage	1 (12.5)	1 (20.0)	1 (25)	0	0 (0)
Ph pos	2 (25.0)	1 (20.0)	1 (50)	0	1 (33.3)
Cytogenetics					
Low risk^a^	3 (3)	3 (3.4)	2 (5)	1 (2.1)	0
Intermediate risk^a^	49 (48.5)	43 (49.4)	23 (57.5)	20 (42.6)	6 (42.9)
High risk^a^	36 (35.6)	29 (33.3)	10 (25)	19 (40.4)	7 (50)
Unknown	13 (12.9)	12 (13.8)	5 (12.5)	7 (14.9)	1 (7.1)
Molecular biology					
BCR-ABL mutated	2/96 (2.1)	1/82 (1.2)	1/37 (2.6)	0/44	1/14 (7.1)
AF4-MLL mutated	1/6 (16.7)	0/3	0/2	0/1	1/3 (33.3)
AML1 ETO mutated	2/91 (2.2)	2/80 (2.5)	1/36 (2.8)	1/44 (2.3)	0/11
FLT3-ITD mutated	21/91 (23.1)	19/80 (23.8)	12/36 (33.3)	7/44 (15.9)	2/11 (18.2)
CEBPA mutated	1/90 (1.1)	1/79 (1.3)	1/36 (2.8)	0/43	0/11
NPM1 mutated	24/90 (26.7)	21/79 (26.6)	15/36 (41.7)	6/43 (14)	3/11 (27.3)
MLL-PTD mutated	1/90 (1.1)	1/79 (1.3)	1/36 (2.8)	0/43	0/11
Sorror score					
0	39 (38.6)	33 (37.9)	13 (32.5)	20 (42.6)	6 (42.9)
1	43 (42.6)	39 (44.8)	19 (47.5)	20 (42.6)	4 (28.6)
2	11 (10.9)	10 (11.5)	5 (12.5)	5 (10.6)	1 (7.1)
3	7 (6.9)	5 (5.7)	3 (7.5)	2 (4.3)	2 (14.3)
>3	1 (1.0)	0 (0)	0 (0)	0 (0)	1 (7.1)
Blasts PB % at enrollement	30 (0–100)	28.5 (0–100)	40 (0–95)	21 (0–100)	70 (4–100)
Platelets ×10^9^/L at enrollement	34 (3–471)	36 (3–471)	35 (3–327)	43 (6–471)	22 (10–165)
Ferritin ng/mL at enrollement	1757 (25–7745)	1740 (122–6853)	1836 (122–6853)	1700 (460–5565)	1914 (25–7745)
Albumin, g/L at enrollement	42 (12–63)	42 (12–63)	43 (12–60.2)	41 (27–63)	40 (30–44.5)
Donor type^b^					
URD	48 (55.2)	48 (55.2)	25 (62.5)	23 (48.9)	–
10/10 matched	26 (54.2)	26 (54.2)	11 (44.0)	15 (65.2)	–
mis-matched	17 (35.4)	17 (35.4)	12 (48.0)	5 (21.7)	–
CB	6 (6.9)	6 (6.9)	11 (27.5)	2 (4.3)	–
Haploidentical	33 (37.9)	33 (37.9)	4 (10.0)	22 (46.8)	–
Female donor for male recipient^b^	18 (20.7)	18 (20.7)	6 (15.0)	12 (25.5)	–
HSC source^b^					
PBSC	44 (50.6)	44 (50.6)	25 (62.5)	19 (40.4)	–
BM	37 (42.5)	37 (42.5)	11 (27.5)	26 (55.3)	–
CB	6 (6.9)	6 (6.9)	4 (10.0)	2 (4.3)	–
CMV serostatus^b^					
R CMV+/D CMV+	42 (51.2)	42 (51.2)	19 (48.7)	23 (53.5)	–
R CMV+/D CMV−	30 (36.6)	30 (36.6)	15 (38.5)	15 (34.9)	–
R CMV-/D CMV+	6 (7.3)	6 (7.3)	3 (7.7)	3 (7)	–
R CMV−/D CMV−	4 (4.9)	4 (4.9)	2 (5.1)	2 (4.7)	–
Conditioning regimen^b^					
TBF-full	82 (94.3)	82 (94.3)	37 (92.5)	45 (95.7)	–
TBF- reduced intensity	4 (4.6)	4 (4.6)	2 (5.0)	2 (4.3)	–
Other	1 (1.1)	1 (1.1)	1 (2.5)	0	–

*HSCT* hematopoietic stem cell transplantation, *URD* unrelated donor, *CB* cord blood, *HSC* hematopoietic stem cell source, *PBSC* peripheral blood stem cells, *BM* bone marrow, *CMV* cytomegalovirus, *R* recipient, *D* donor, *TBF* thiotepa, busulfan, fludarabine.

^a^AML patients with t(inv16), t(8:21) were cytogenetically considered as low-risk patients; those with normal karyotype, trisomy of chromosome (chr) 8, trisomy of chr 21, del 12(p13), additional chr 16 and 1 as intermediate and, finally, those showing complex karyotype, monosomy or other abnormalities of chr. 5 and 7, 17p-, as high risk; ALL patients with t(9:22), t(4;11) and abnormalities of 11 were considered high-risk cytogenetics.

^b^Only patients transplanted.

The median times to neutrophil (neutrophils greater than 0.5 × 10^9^/L) and platelet (platelets greater than 20 ×10^9^/L) engraftment were 20 and 25 days while the cumulative incidence was 78% and 71%, respectively, at 100 days.

Overall, with a median follow up of 9 months (range 0.7–93) for the patients enrolled into the study and 74 months (range: 11–93) for the living patients, 89 died: 59 due to disease recurrence while 30 deaths were related to transplant. Among the 87 patients who were transplanted, 76 patients died, 46 because of disease recurrence and 30 died due to NRM. Among these latter, 11 patents died because of infection (10/11 bacterial, 1/11 fungal infection), 5 because of GVHD, 11 of multi-organ failure, 2 after an acute respiratory distress, and one of hyperacute hemolytic event.

**Fig. 2:**
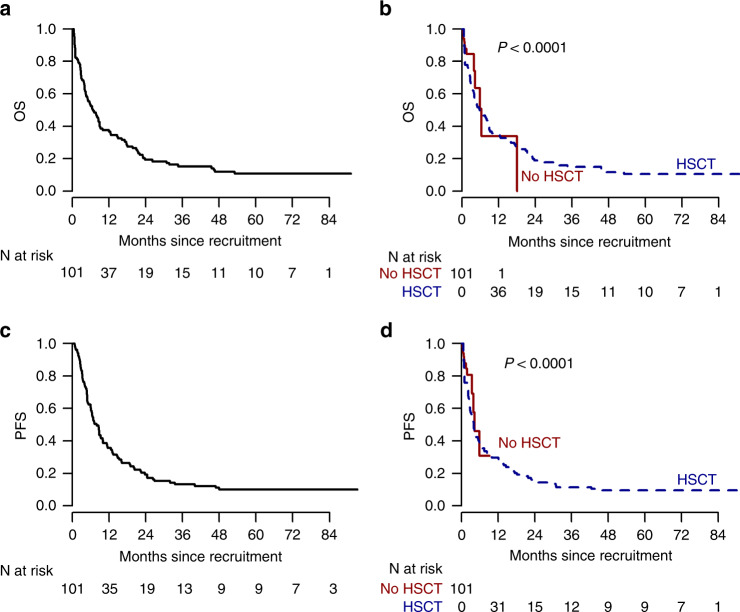
Overall survival and progression-free survival of the entire study population. Overall survival of the overall population (**a**); Simon–Makuch survival curves comparing the transplant vs not transplant cohorts (**b**). Progression-free survival of the overall population (**c**); Simon-Makuch progression-free survival curves comparing the transplant vs not transplant cohorts (**d**). OS overall survival, PFS progression-free survival.

**Fig. 3:**
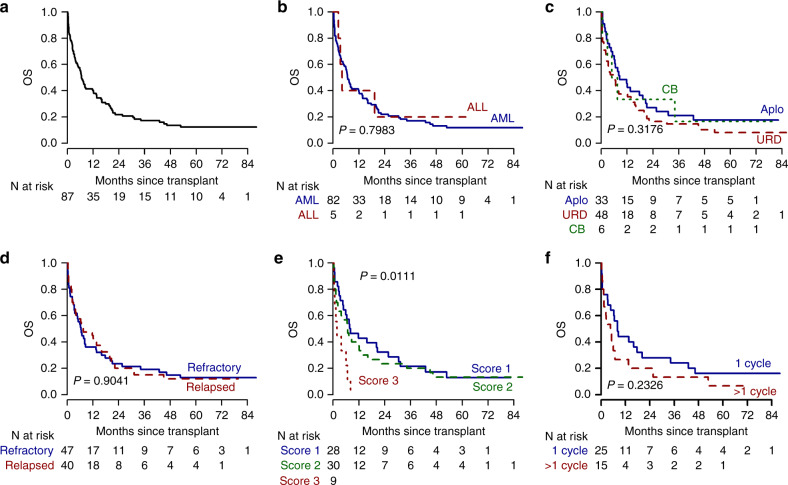
Overall survival and progression-free survival of the transplant cohort. Overall survival of the transplant cohort (**a**); overall survival according to disease: ALL vs AML (**b**), to donor type (**c**), to disease history: relapsed vs refractory (**d**), and to cIBMTR score (**e**); overall survival, in the refractory cohort, according to the number of pre-transplant cycles of chemotherapy (**f**). OS overall survival, ALL acute lymphoblastic leukemia, AML acute Myeloid Leukemia, cIBMTR center of International Bone Marrow Transplantation Registry.

The OS of the overall population was 38% and 19% at 1 and 2 years, respectively (Fig. 2a); the OS in the transplant vs no transplant cohort is reported in Fig. 2b; in the transplant cohort OS was 40% and 22% at 1 and 2 years (Fig. 3a), and no significant differences were found according to disease (Fig. 3b), donor type (Fig. 3c) and disease history (refractory vs relapsed) (Fig. 3d) and to CIBMTR score (Fig. 3e). In the refractory cohort, OS shows higher estimates for patients being transplanted after failing only one cycle of chemotherapy, without reaching however the statistical significance (Fig. 3f).

The PFS at two years was 19% and 17% for the overall population and for the transplant cohort (Fig. 2c, d).

**Fig. 4:**
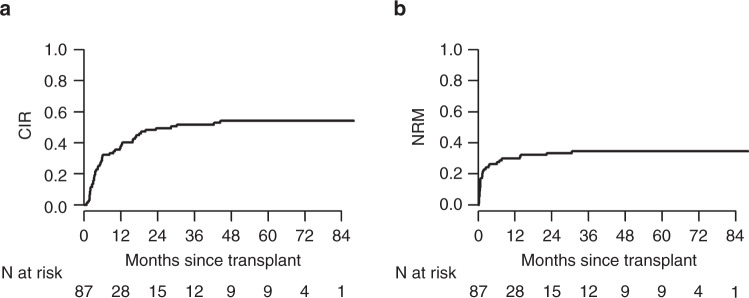
Cumulative incidence of relapse and non-relapse mortality of the transplant cohort. **A** The cumulative incidence of relapse in the transplant cohort; **B** the non-relapse mortality of the transplant cohort. CIR cumulative incidence of relapse, NRM non-relapse mortality.

At 1 and 2 years after transplant, the CIR was 38% and 49% while the NRM was 30% and 33%, respectively (Fig. 4). OS and NRM were univariately associated with Sorror comorbidity index (Table 2) while CIR was slightly lower in refractory compared to relapsed patients (Table 2 and Supplementary Fig 3). Characteristics of long-term survivors are shown in Table 3.

**Table 2:** Univariate analysis on transplanted patients.

	OS HR (95% CI)	OS *P* value	CIR HR (95% CI)	CIR *P* value	NRM HR (95% CI)	NRM *P* value
Age (years)	1.01 (1–1.03)	0.0907	0.99 (0.97–1.01)	0.38	1.03 (1–1.06)	0.077
Sex						
Female	1		1		1	
Male	1.19 (0.76–1.87)	0.4504	0.7 (0.4–1.23)	0.21	1.64 (0.79–3.4)	0.18
Previous autologous transplant	0.98 (0.45–2.14)	0.9655	0.81 (0.32–2.1)	0.67	1.08 (0.35–3.34)	0.9
Diagnosis						
Acute Myeloid Leukemia	1		1		1	
Acute Lymphoblastic Leukemia	0.88 (0.32–2.4)	0.7987	1.51 (0.38–6.09)	0.56	0.49 (0.07–3.27)	0.46
Clinical history						
Primary refractory	1		1		1	
Relapsed	0.97 (0.62–1.53)	0.9058	**1.88 (1.06–3.34)**	**0.03**	0.53 (0.25–1.13)	0.1
Cytogenetics						
low risk	3.57 (1.08–11.82)	0.037	0 (0–0)	<0.0001	7.7 (3.03–19.56)	<.0001
Intermediate risk	1		1		1	
High risk	1.36 (0.82–2.26)	0.2299	0.78 (0.4–1.51)	0.46	1.84 (0.82–4.12)	0.14
Unknown	1.38 (0.7–2.72)	0.3506	1.02 (0.44–2.37)	0.96	1.46 (0.45–4.69)	0.53
Molecular biology						
FLT3-ITD mutated	1.25 (0.72–2.17)	0.4243	1.82 (0.87–3.83)	0.11	0.59 (0.23–1.49)	0.27
NPM1 mutated	0.84 (0.48–1.45)	0.5266	1.29 (0.65–2.56)	0.47	0.64 (0.27–1.51)	0.31
Sorror score						
0	1		1		1	
1	**1.99 (1.18–3.34)**	**0.0094**	0.88 (0.47–1.64)	0.69	**3.41 (1.27–9.18)**	**0.015**
2	**2.4 (1.13–5.1)**	**0.0225**	0.98 (0.41–2.32)	0.96	3.12 (0.85–11.52)	0.088
3	**4.93 (1.83–13.26)**	**0.0016**	0.25 (0.04–1.69)	0.15	**11.04 (2.73–44.68)**	**0.0008**
Blasts in the peripheral blood (%)	1 (0.99–1.01)	0.4927	1 (0.99-1.01)	0.94	1 (0.99–1.01)	0.87
Platelets ×10^*9*^*/L*	1 (1–1)	0.6866	1 (1-1)	0.86	1 (1–1)	0.99
Ferritin ng/mL	1 (1–1)	0.019	1 (1-1)	0.7	1 (1–1)	0.38
Albumin mg/dL	0.99 (0.98–1.01)	0.3046	1 (0.99–1.02)	0.56	0.99 (0.97–1.01)	0.34
Donor type						
URD	1.43 (0.89–2.32)	0.1404	0.83 (0.46–1.5)	0.54	1.78 (0.84–3.8)	0.13
CB	1.1 (0.42–2.86)	0.8467	1.42 (0.46–4.43)	0.54	0.62 (0.07–5.27)	0.67
Haploidentical	1		1		1	
Female donor for male recipient	0.82 (0.47–1.44)	0.4936	0.68 (0.33–1.42)	0.3	1.11 (0.5–2.46)	0.79
HSC source						
BM	1		1		1	
PBSC	1.22 (0.77–1.96)	0.3971	0.75 (0.42–1.36)	0.35	1.58 (0.77–3.27)	0.21
CB	0.99 (0.39–2.56)	0.9901	1.37 (0.44–4.2)	0.59	0.56 (0.07–4.69)	0.6
CMV serostatus						
R CMV+/D CMV+	1		1		1	
R CMV+/D CMV−	0.9 (0.54–1.48)	0.6675	0.99 (0.54–1.81)	0.96	0.86 (0.38–1.95)	0.71
R CMV−/D CMV+	0.79 (0.31–2.01)	0.6225	1.37 (0.46–4.04)	0.57	0.42 (0.06–3.11)	0.4
R CMV−/D CMV−	0.89 (0.27–2.89)	0.8444	0.4 (0.04–3.9)	0.43	1.68 (0.38–7.4)	0.49
Acute GvHD^a^	1.24 (0.74–2.05)	0.41	0.74 (0.40–1.37)	0.34	1.61 (0.70–3.73)	0.26
Chronic GvHD^a^	1.08 (0.50–2.33)	0.84	1.22 (0.50–2.99)	0.66	0.95 (0.26–3.48)	0.94

Bold values indicate statistically significant results.

*OS* overall survival, *HR* hazard risk, *CIR* cumulative incidence of relapse, *NRM* non-relapse mortality, *URD* unrelated donor, *CB* cord blood, *HSC* hematopoietic stem cell source, *BM* bone marrow, *PBSC* peripheral blood stem cells, *CMV* cytomegalovirus, *R* recipient, *D* donor, *TBF* thiotepa, busulfan, fludarabine, *GVHD* graft-versus-host disease.

^a^Time-dependent variable.

**Table 3:** Characteristics of long-term survivors.

	Alive at 3 years (*n* = 15)	Alive at 5 years (*n* = 10)
Age (years), median (range)	38 (25–68)	51.5 (26–68)
Sex		
Female	7 (46.7)	5 (50)
Male	8 (53.3)	5 (50)
Time from diagnosis to inclusion	49 (0–225)	43.5 (0–198)
Time from enrollment to transplant	25 (7–155)	19.5 (7–155)
Previous auto HSCT	1 (6.7)	0
Clinical history		
Primary refractory	9 (60)	6 (60)
Relapsed	6 (40)	4 (40)
Diagnosis		
Acute Myeloid Leukemia	14 (93.3)	9 (90.0)
de novo	11 (78.6)	7 (77.8)
Secondary	3 (21.4)	2 (22.2)
Therapy-related	0	0
Acute Lymphoblastic Leukemia	1 (6.7)	1 (10.0)
de novo	1 (100)	1 (100)
Secondary	0	0
B-lineage	1 (100)	1 (100)
T- lineage	0	0
Ph pos	1 (100)	1 (100)
Cytogenetics		
Low risk	0	0
Intermediate risk	9 (60)	6 (60)
High risk	5 (33.3)	3 (30)
Unknown	1 (6.7)	1 (10)
Molecular biology		
BCR-ABL mutated	1/13 (7.7)	1 (11.1)
AF4-MLL mutated	0/1	0
AML1 ETO mutated	0/12	0
FLT3-ITD mutated	2/12 (16.7)	1 (12.5)
CEBPA mutated	0/12	0
NPM1 mutated	4/12 (33.3)	3 (37.5)
MLL-PTD mutated	0/12	0
Sorror score		
0	11 (73.3)	8 (80)
1	4 (26.7)	2 (20)
2	0 (0)	0 (0)
3	0 (0)	0 (0)
>3	0 (0)	0 (0)
Blasts PB % at enrollment	15 (0–60)	23.5 (0–60)
Platelets ×10^*9*^*/L at enrollment*	44 (3–242)	40 (12–242)
Ferritin ng/mL at enrollment	1480 (624–3019)	1031 (624–2556)
Albumin, g/L at enrollment	4.6 (3.2–45)	4.4 (3.5–45)
Donor type		
URD	7 (46.7)	4 (40)
10/10 matched	5 (71.4)	4 (100)
mis-matched	2 (28.6)	0
CB	1 (6.7)	1 (10)
Haploidentical	7 (46.7)	5 (50)
Female donor for male recipient	4 (26.7)	2 (20)
HSC source		
PBSC	6 (40)	4 (40)
BM	8 (53.3)	5 (50)
CB	1 (6.7)	1 (10)
CMV serostatus		
R CMV+/D CMV+	6 (42.9)	3 (33.3)
R CMV+/D CMV−	6 (42.9)	4 (44.4)
R CMV−/D CMV+	1 (7.1)	1 (11.1)
R CMV−/D CMV−	1 (7.1)	1 (11.1)
Acute GvHD	7 (46.7)	4 (40)
Chronic GvHD	2 (13.3)	1 (10)

*HSCT* hematopoietic stem cell transplantation, *PB* peripheral blood, *HSC* hematopoietic stem cell source, *PBSC* peripheral blood stem cells, *BM* bone marrow, *URD* unrelated donor, *CB* cord blood, *CMV* cytomegalovirus, *R* recipient, *D* donor, *GVHD* graft-versus-host disease.

Thirty-seven patients experienced acute GVHD (14 grade I, 13 grade II, 6 grade III, 4 grade IV) while only 10 developed chronic GVHD (4 mild, 4 moderate, 2 severe).

## Discussion

The present study shows the long-term clinical outcome of patients with R/R acute leukemias treated with a myeloablative transplant from alternative donors in an *intention-to-transplant* trial.

The choice of a MAC based on a double alkylating agents schedule, in our prospective study did not improve significantly the leukemia control and the NRM proved remarkably high. In a retrospective Registry study, focused on sibling transplants [21], TBF regimen was associated with 2-yr NRM of 27% versus 16% in the busulfan-fludarabine cohort and lower risk of disease recurrence.

The high NRM in this study (33% at two years) can be attributed to the highly intensive conditioning regimen using thiotepa at the dose of 10 mg/kg and busulfan at 9.6 mg/mg (T_2_B_3_F) in a heavily pretreated patients, whose median age at transplant was 54 years. Rodriguez-Arbolì et al. [22]. showed lower NRM after TBF regimen in a patient population (with almost 50% patients being in CR) with an age distribution similar to that reported in the present study. However, lower doses of either busulfan or thiotepa were given to a significant proportion of patients [22]. Reduction of doses were also reported in the haplo-HSCT setting [23], where the reduction of thiotepa dose significantly spared toxicity without loosing the disease control.

The sequential approach [24] was designed for R/R acute leukemias with the aim to guarantee an high antileukemic activity with chemo/radiotherapy soon before conditioning, an immunosuppressive effect allowing engraftment after a RIC, which can limit NRM, and finally preemptive donor lymphocyte infusions to elicit early post-transplant graft-versus-malignancy effect. No robust data are still available to investigate if a MAC approach is better than a sequential-RIC regimen in this setting. A retrospective registry analysis [22] compared 1018 patients younger than 50 years transplanted with active disease after either a MAC regimen (TBI or busulfan) or FLAMSA-RIC (with TBI or with chemotherapy) showing better OS and lower NRM for FLAMSA-chemotherapy. On the contrary, a multicenter retrospective analysis reported by Decroocq [25] and the analysis conducted by the Acute Leukemia Working Party of the EBMT [26] did not find any differences in the transplant outcome between myeloablative conditioning or treosulfan-fludarabine regimen with the sequential FLAMSA-RIC approach.

Several attempts to modify the original FLAMSA-RIC schedule have been proposed to increase the efficacy of transplant in high-risk AML using either clofarabine, busulfan, treosulfan or melphalan [27–29] but not definitive data are available at the moment to establish the best combination. The only available prospective controlled study [30] reported the absence of survival advantage after intensification of FLAMSA-busulfan regimen in high-risk AML and myelodysplastic syndromes.

We have to acknowledge several major limitations of this study including the non-randomized design and the sample size which can limit the likelihood to capture the effect of already validated prognostic factors such as cytogenetics, number of courses of pretransplant treatments, disease burden at conditioning and the pre-transplant history of disease [31, 32].

The study we reported here did not include any requirements in term of type and number of pre-transplant cycles of chemotherapy and all patients were transplanted with active disease. Proceeding to allo-HSCT trying or not to obtain a CR with either chemotherapy regimens, or novel drugs or targeted therapies even with subsequent trials, is still an open question, particularly in AML, [1, 33–42] NCT024615357 ETAL-3-ASAP trial is ongoing) while in ALL the use of immunotherapy before transplant is recommended [5, 6, 43]

Since only 14 patients did not undergo allo-HSCT we were not able to demonstrate the benefit of transplant over the non-transplant cohort However, based on the survival plot reported in Fig. 2b it is temptive to speculate that allo-HSCT may provide a limited beneficial effect on long term survival in these patients is confirmed, even after correcting for the time to transplant bias.

GVHD was not the leading cause of NRM in this setting of patients. Instead, bacterial infections had a leading role in the early post-transplant NRM [44], especially after intensive MAC regimen.

Despite the unsatisfactory results, a major strength of this study is represented by its prospective design in opening the chance to access alternative donor transplant in the R/R setting, the uniform criteria adopted for patients’ selection, the possibility to use any possible alternative donors and the unique conditioning regimen.

The characteristics of the patient population here reported are closer to the real life more than to selective clinical trials, limiting significantly the potential positive selection bias of a non-randomized design. For this reason, although the definition of refractoriness in the study did not match perfectly the criteria proposed by Ferguson et al. [45], it cannot surprise that the patients here enrolled fully mirror the fate of patients not achieving a CR after two courses of chemotherapy (REF2 population elsewhere described [45]).

The crucial factors dictating the poor prognosis of the patients here reported were the use of a very intensive regimen, the truly refractory status and pretreatment burden, and, even more importantly, the absence of a pre-planned strategy to prevent relapse included in the study.

At this stage, we believe that the need for a high-intensity conditioning regimen for transplant in active disease should be integrated with approaches aimed at lowering post-transplant relapse risk such as prophylactic modified or unmodified DLIs, targeted drugs maintenance, immunosuppression modulation [46], as indeed envisaged in the original conceptualization of the sequential approach [47] and prospectively tested in this setting.

## Supplementary information

Supplementary material

## Data Availability

The datasets generated and analyzed during the current study are available from the corresponding author on reasonable request.

## References

[1] Thol F, Schlenk RF, Heuser M, Ganser A (2015). How I treat refractory and early relapsed acute myeloid leukemia. Blood.

[2] Othus M, Appelbaum FR, Petersdorf SH, Kopecky KJ, Slovak M, Nevill T (2015). Fate of patients with newly diagnosed acute myeloid leukemia who fail primary induction therapy. Biol Blood Marrow Transplant.

[3] Duval M, Klein JP, He W, Cahn JY, Cairo M, Camitta BM (2010). Hematopoietic stem-cell transplantation for acute leukemia in relapse or primary induction failure. J Clin Oncol.

[4] Paul S, Rausch CR, Nasnas PE, Kantarjian HJE (2019). No title. Clin Adv Hematol Oncol.

[5] Kantarjian H, Stein A, Gökbuget N, Fielding AK, Schuh AC, Ribera J-M (2017). Blinatumomab versus chemotherapy for advanced acute lymphoblastic leukemia. N Engl J Med.

[6] Kantarjian HM, DeAngelo DJ, Stelljes M, Martinelli G, Liedtke M, Stock W (2016). Inotuzumab ozogamicin versus standard therapy for acute lymphoblastic leukemia. N Engl J Med.

[7] Todisco E, Ciceri F, Boschini C, Giglio F, Bacigalupo A, Patriarca F, et al. Factors predicting outcome after allogeneic transplant in refractory acute myeloid leukemia: a retrospective analysis of Gruppo Italiano Trapianto di Midollo Osseo (GITMO). Bone Marrow Transplant. 2017;52:955–61. 10.1038/bmt.2016.325 28067875

[8] Döhner H, Estey EH, Amadori S, Appelbaum FR, Büchner T, Burnett AK (2010). Diagnosis and management of acute myeloid leukemia in adults: recommendations from an international expert panel, on behalf of the European LeukemiaNet. Blood.

[9] Döhner H, Estey E, Grimwade D, Amadori S, Appelbaum FR, Büchner T (2017). Diagnosis and management of AML in adults: 2017 ELN recommendations from an international expert panel. Blood.

[10] Sanz J, Boluda JCH, Martín C, González M, Ferrá C, Serrano D (2012). Single-unit umbilical cord blood transplantation from unrelated donors in patients with hematological malignancy using busulfan, thiotepa, fludarabine and ATG as myeloablative conditioning regimen. Bone Marrow Transplant.

[11] Sanz J, Picardi A, Hernández Boluda JC, Martín C, Ferrá C, Nozzoli C (2013). Impact of graft-versus-host disease prophylaxis on outcomes after myeloablative single-unit umbilical cord blood transplantation. Biol Blood Marrow Transplant.

[12] Bartolomeo PDI, Santarone S, De Angelis G, Picardi A, Cudillo L, Cerretti R (2013). Haploidentical, unmanipulated, G-CSF-primed bone marrow transplantation for patients with high-risk hematologic malignancies. Blood.

[13] Raiola AM, Dominietto A, Ghiso A, Di Grazia C, Lamparelli T, Gualandi F (2013). Unmanipulated haploidentical bone marrow transplantation and posttransplantation cyclophosphamide for hematologic malignancies after myeloablative conditioning. Biol Blood Marrow Transplant.

[14] Bacigalupo A, Dominietto A, Ghiso A, Di Grazia C, Lamparelli T, Gualandi F (2015). Unmanipulated haploidentical bone marrow transplantation and post-transplant cyclophosphamide for hematologic malignanices following a myeloablative conditioning: an update. Bone Marrow Transplant.

[15] Arcese W, Picardi A, Santarone S, De Angelis G, Cerretti R, Cudillo L (2015). Haploidentical, G-CSF-primed, unmanipulated bone marrow transplantation for patients with high-risk hematological malignancies: An update. Bone Marrow Transplant.

[16] Peters C, Dalle J-H, Locatelli F, Poetschger U, Sedlacek P, Buechner J (2020). Total body irradiation or chemotherapy conditioning in childhood ALL: a multinational, randomized, noninferiority phase III study. J Clin Oncol.

[17] Khimani F, Dutta M, Faramand R, Nishihori T, Perez AP, Dean E (2021). Impact of total body irradiation-based myeloablative conditioning regimens in patients with acute lymphoblastic leukemia undergoing allogeneic hematopoietic stem cell transplantation: systematic review and meta-analysis. Transpl Cell Ther.

[18] Kebriaei P, Anasetti C, Zhang MJ, Wang HL, Aldoss I, de Lima M (2018). Intravenous busulfan compared with total body irradiation pretransplant conditioning for adults with acute lymphoblastic leukemia. Biol Blood Marrow Transplant.

[19] Dholaria B, Labopin M, Angelucci E, Tischer J, Arat M, Ciceri F (2021). Improved outcomes of haploidentical hematopoietic cell transplantation with total body irradiation-based myeloablative conditioning in acute lymphoblastic leukemia. Transpl Cell Ther..

[20] Luznik L, O’Donnell PV, Symons HJ, Chen AR, Leffell MS, Zahurak M (2008). HLA-haploidentical bone marrow transplantation for hematologic malignancies using nonmyeloablative conditioning and high-dose, posttransplantation cyclophosphamide. Biol Blood Marrow Transplant.

[21] Saraceni F, Labopin M, Hamladji RM, Mufti G, Socié G, Shimoni A (2018). Thiotepa-busulfan-fludarabine compared to busulfan-fludarabine for sibling and unrelated donor transplant in acute myeloid leukemia in first remission. Oncotarget..

[22] Rodríguez-Arbolí E, Labopin M, Tischer J, Brecht A, Ganser A, Finke J (2020). FLAMSA-based reduced-intensity conditioning versus myeloablative conditioning in younger patients with relapsed/refractory acute myeloid leukemia with active disease at the time of allogeneic stem cell transplantation: an analysis from the acute leukemia. Biol Blood Marrow Transplant.

[23] Duléry R, Ménard AL, Chantepie S, El-Cheikh J, François S, Delage J (2018). Sequential conditioning with thiotepa in T cell-replete hematopoietic stem cell transplantation for the treatment of refractory hematologic malignancies: comparison with matched related, haplo-mismatched, and unrelated donors. Biol Blood Marrow Transplant.

[24] Schmid C, Schleuning M, Ledderose G, Tischer J, Kolb HJ (2005). Sequential regimen of chemotherapy, reduced-intensity conditioning for allogeneic stem-cell transplantation, and prophylactic donor lymphocyte transfusion in high-risk acute myeloid leukemia and myelodysplastic syndrome. J Clin Oncol.

[25] Decroocq J, Itzykson R, Vigouroux S, Michallet M, Yakoub-Agha I, Huynh A (2018). Similar outcome of allogeneic stem cell transplantation after myeloablative and sequential conditioning regimen in patients with refractory or relapsed acute myeloid leukemia: a study from the Société Francophone de Greffe de Moelle et de Thérapie Cellula. Am J Hematol.

[26] Saraceni F, Labopin M, Brecht A, Kröger N, Eder M, Tischer J (2019). Fludarabine-treosulfan compared to thiotepa-busulfan-fludarabine or FLAMSA as conditioning regimen for patients with primary refractory or relapsed acute myeloid leukemia: a study from the Acute Leukemia Working Party of the European Society for Blood and Marrow Transplantation (EBMT). J Hematol Oncol.

[27] Heinicke T, Labopin M, Polge E, Stelljes M, Ganser A, Tischer J (2021). Evaluation of six different types of sequential conditioning regimens for allogeneic stem cell transplantation in relapsed/refractory acute myelogenous leukemia – a study of the Acute Leukemia Working Party of the EBMT. Leuk Lymphoma.

[28] Jondreville L, Roos-Weil D, Uzunov M, Boussen I, Grenier A, Norol F (2021). FLAMSA-busulfan-melphalan as a sequential conditioning regimen in HLA-matched or haploidentical hematopoietic stem cell transplantation for high-risk myeloid diseases. Transpl Cell Ther..

[29] Le Bourgeois A, Labopin M, Marçais A, de Latour RP, Blaise D, Chantepie S (2020). Sequential allogeneic hematopoietic stem cell transplantation for active refractory/relapsed myeloid malignancies: results of a reduced-intensity conditioning preceded by clofarabine and cytosine arabinoside, a retrospective study on behalf of the SFGM-TC. Ann Hematol..

[30] Craddock C, Jackson A, Loke J, Siddique S, Hodgkinson A, Mason J (2021). Augmented reduced-intensity regimen does not improve postallogeneic transplant outcomes in acute myeloid leukemia. J Clin Oncol.

[31] Craddock C, Labopin M, Pillai S, Finke J, Bunjes D, Greinix H (2011). Factors predicting outcome after unrelated donor stem cell transplantation in primary refractory acute myeloid leukaemia. Leukemia..

[32] Todisco E, Ciceri F, Oldani E, Boschini C, Micò C, Vanlint MT (2013). The CIBMTR score predicts survival of AML patients undergoing allogeneic transplantation with active disease after a myeloablative or reduced intensity conditioning: a retrospective analysis of the Gruppo Italiano Trapianto di Midollo Osseo. Leukemia..

[33] Wattad M, Weber D, Döhner K, Krauter J, Gaidzik VI, Paschka P (2017). Impact of salvage regimens on response and overall survival in acute myeloid leukemia with induction failure. Leukemia..

[34] Schlenk RF, Döhner K, Mack S, Stoppel M, Király F, Götze K (2010). Prospective evaluation of allogeneic hematopoietic stem-cell transplantation from matched related and matched unrelated donors in younger adults with high-risk acute myeloid leukemia: German-Austrian trial AMLHD98A. J Clin Oncol.

[35] Ivanoff S, Gruson B, Chantepie SP, Lemasle E, Merlusca L, Harrivel V (2013). 5-Azacytidine treatment for relapsed or refractory acute myeloid leukemia after intensive chemotherapy. Am J Hematol.

[36] Sierra J, Storer B, Hansen JA, Bjerke JW, Martin PJ, Petersdorf EW (1997). Transplantation of marrow cells from unrelated donors for treatment of high-risk acute leukemia: The effect of leukemic burden, donor HLA-matching, and marrow cell dose. Blood..

[37] Konopleva M, Pollyea DA, Potluri J, Chyla B, Hogdal L, Busman T (2016). Efficacy and biological correlates of response in a phase II study of venetoclax monotherapy in patients with acute myelogenous leukemia. Cancer Discov.

[38] Rashidi A, Weisdorf DJ, Bejanyan N (2018). Treatment of relapsed/refractory acute myeloid leukaemia in adults. Br J Haematol.

[39] Steckel NK, Groth C, Mikesch JH, Trenschel R, Ottinger H, Kordelas L (2018). High-dose melphalan-based sequential conditioning chemotherapy followed by allogeneic haematopoietic stem cell transplantation in adult patients with relapsed or refractory acute myeloid leukaemia. Br J Haematol.

[40] Schroeder T, Wegener N, Lauseker M, Rautenberg C, Nachtkamp K, Schuler E (2019). Comparison between upfront transplantation and different pretransplant cytoreductive treatment approaches in patients with high-risk myelodysplastic syndrome and secondary acute myelogenous leukemia. Biol Blood Marrow Transplant.

[41] Short NJ, Rafei H, Daver N, Hwang H, Ning J, Jorgensen JL (2020). Prognostic impact of complete remission with MRD negativity in patients with relapsed or refractory AML. Blood Adv.

[42] Montesinos P, Bergua J, Infante J, Esteve J, Guimaraes JE, Sierra J (2020). Correction to: update on management and progress of novel therapeutics for R/R AML: an Iberian expert panel consensus. Ann Hematol.

[43] Gökbuget N, Stanze D, Beck J, Diedrich H, Horst HA, Hüttmann A (2012). Outcome of relapsed adult lymphoblastic leukemia depends on response to salvage chemotherapy, prognostic factors, and performance of stem cell transplantation. Blood..

[44] Girmenia C, Bertaina A, Piciocchi A, Perruccio K, Algarotti A, Busca A, et al. Incidence, risk factors and outcome of pre-engraftment gram-negative bacteremia after allogeneic and autologous hematopoietic stem cell transplantation: an Italian Prospective Multicenter Survey. Clin Infect Dis. 2017;65:1884–96. 10.1093/cid/cix690 29020286

[45] Ferguson P, Hills RK, Grech A, Betteridge S, Kjeldsen L, Dennis M (2016). An operational definition of primary refractory acute myeloid leukemia allowing early identification of patients who may benefit from allogeneic stem cell transplantation. Haematologica..

[46] Loke J, Malladi R, Moss P, Craddock C (2020). The role of allogeneic stem cell transplantation in the management of acute myeloid leukaemia: a triumph of hope and experience. Br J Haematol.

[47] Kolb HJ, Schmid C (2020). The FLAMSA concept—past and future. Ann Hematol..

