# Supplementary material for: Myeloablative conditioning with thiotepa-busulfan-fludarabine does not improve the outcome of patients transplanted with active leukemia: final results of the GITMO prospective trial GANDALF-01
Source: Bone Marrow Transplant. 2022 Apr 12;57(6):949–58. doi: 10.1038/s41409-022-01626-5 (PMC9200637; doi:10.1038/s41409-022-01626-5)

SUPPLEMENTARY MATERIAL

**Myeloablative conditioning with thiotepa-busulfan-fludarabine does not improve the outcome of patients transplanted with active leukemia: final results of the GITMO prospective trial GANDALF-01**

Supplementary tables

Supplementary table 1: Participating centers and number of enrolled patients

| **Center** | **N. of enrolled patients** |
| --- | --- |
| Milano - IRCCS Ospedale San Raffaele | 20 |
| Bologna - IRCCS Azienda Ospedaliero-Universitaria di Bologna | 7 |
| Torino - Azienda Ospedaliero-Universitaria Città della Salute e della Scienza di Torino | 7 |
| Pavia - Fondazione I.R.C.C.S. Policlinico San Matteo | 6 |
| Bergamo - Azienda Socio Sanitaria Territoriale Papa Giovanni XXIII | 5 |
| Roma - AOU Policlinico Umberto 1, Sapienza Università di Roma | 5 |
| Udine - Azienda Sanitaria Universitaria Friuli Centrale (ASU FC) | 5 |
| Alessandria - Azienda Ospedaliera Nazionale Santi Antonio e Biagio e Cesare Arrigo | 4 |
| Genova - Ospedale Policlinico San Martino IRCCS | 4 |
| San Giovanni Rotondo - Fondazione Casa Sollievo della Sofferenza | 3 |
| Orbassano - Azienda Ospedaliera Universitaria San Luigi Gonzaga Università di Torino | 3 |
| Roma - Fondazione PTV - Policlinico Tor Vergata Università degli studi di Roma | 3 |
| Roma - Azienda Ospedaliera San Camillo Forlanini | 3 |
| Pescara - Azienda Sanitaria Locale Presidio Ospedaliero di Pescara | 3 |
| Cuneo - Azienda Ospedaliera Santa Croce e Carle Cuneo | 3 |
| Bari - Azienda Ospedaliero Universitaria Consorziale Policlinico di Bari | 2 |
| Roma - Fondazione Policlinico Universitario Agostino Gemelli IRCCS Università Cattolica del Sacro Cuore | 2 |
| Catania - Azienda Ospedaliero Universitaria Policlinico "G. Rodolico - San Marco" | 2 |
| Modena - Azienda Ospedaliero-Universitaria di Modena | 2 |
| Firenze - AOUC Azienda Ospedaliero-Universitaria Careggi | 2 |
| Cagliari - ASSL Cagliari Ospedale Oncologico Armando Businco | 1 |
| Bolzano - Azienda Sanitaria dell'Alto Adige Ospedale di Bolzano | 1 |
| Milano - ASST Grande Ospedale Metropolitano Niguarda | 1 |
| Palermo - La Maddalena S.p.A. Dipartimento Oncologico di 3° Livello | 1 |
| Milano - Fondazione IRCCS Ca' Granda Ospedale Maggiore Policlinico Università degli Studi di Milano | 1 |
| Taranto - ASL Taranto - Ospedale S. G. Moscati | 1 |
| Monza - Azienda Socio Sanitaria Territoriale (ASST) di Monza Ospedale San Gerardo | 1 |
| Palermo - Azienda Ospedaliera Ospedali Riuniti Villa Sofia-Cervello | 1 |
| Cagliari - ASSL Cagliari - Ospedale Binaghi | 1 |
| Piacenza - Azienda Unità Sanitaria Locale - Ospedale Guglielmo da Saliceto | 1 |
| **Total** | **101** |

Supplementary table 2: Criteria for donor search:

| Algorthm for URD search | As matched as possible (on 10 loci)  < 3 mismatches allowed | Mismatches defined at allelic level [4 digits] through high resolution DNA techniques |
| --- | --- | --- |
|  |  |  |
| Algorithm for CB unit selection | > 4/6 matched CB unit & total nucleated cells > 2.5 x10^7^/kg | Mismatches in Class I – HLA-A and –B– defined at antigenic level [2 digits] by means of serology or low-resolution DNA techniques and DRB1 at allelic level [4 digits] through high resolution DNA techniques |
|  |  |  |
| Algorithm of haploidentical donor selection | Hierarchy:  CMV serostatus: for CMV seronegative recipients, a CMV seronegative donor  Mothers  red blood compatible pairs  NK alloreactive donor vs host |  |

Supplementary figures:

Suppl. Figure 1: OS of the overall population (Panel A) and Simon-Makuch survival curves comparing the transplant vs not transplant cohorts (panel B) using diagnosis of refractoriness/resistance as starting point


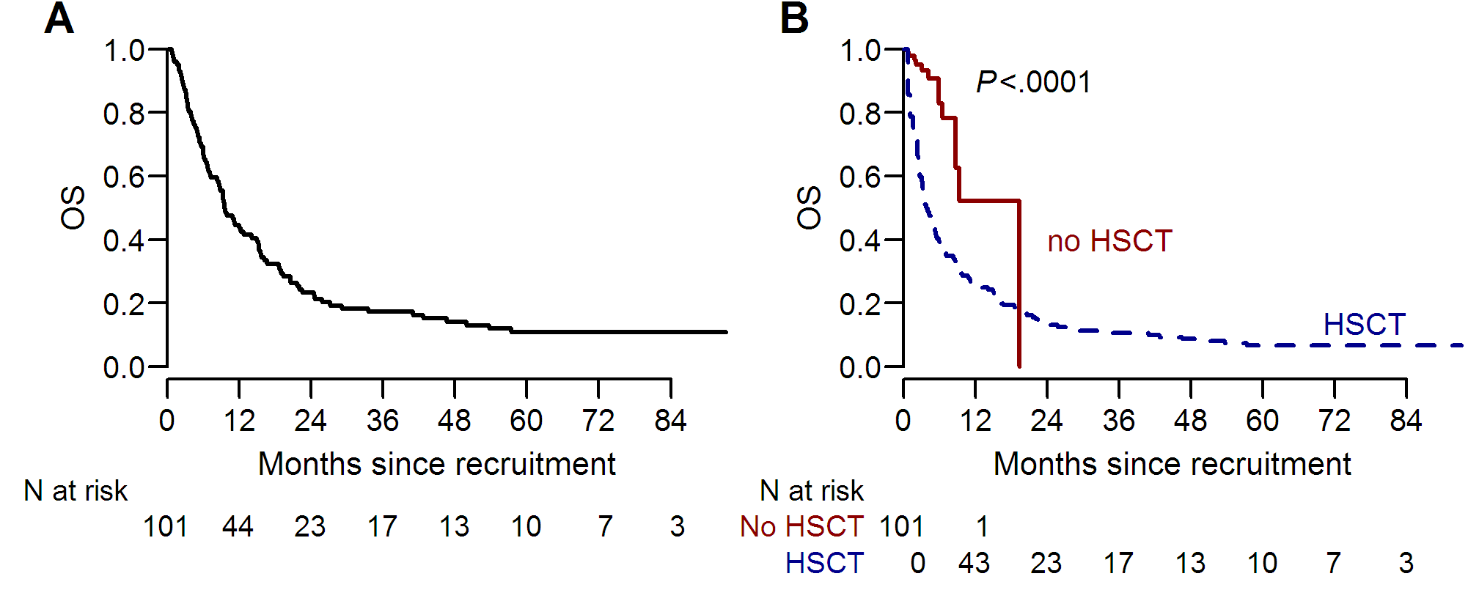


Supplementary Figure 2: PFS of the overall population (Panel A) and Simon-Makuch survival curves comparing the transplant vs not transplant cohorts (panel B) using diagnosis of refractoriness/resistance as starting point


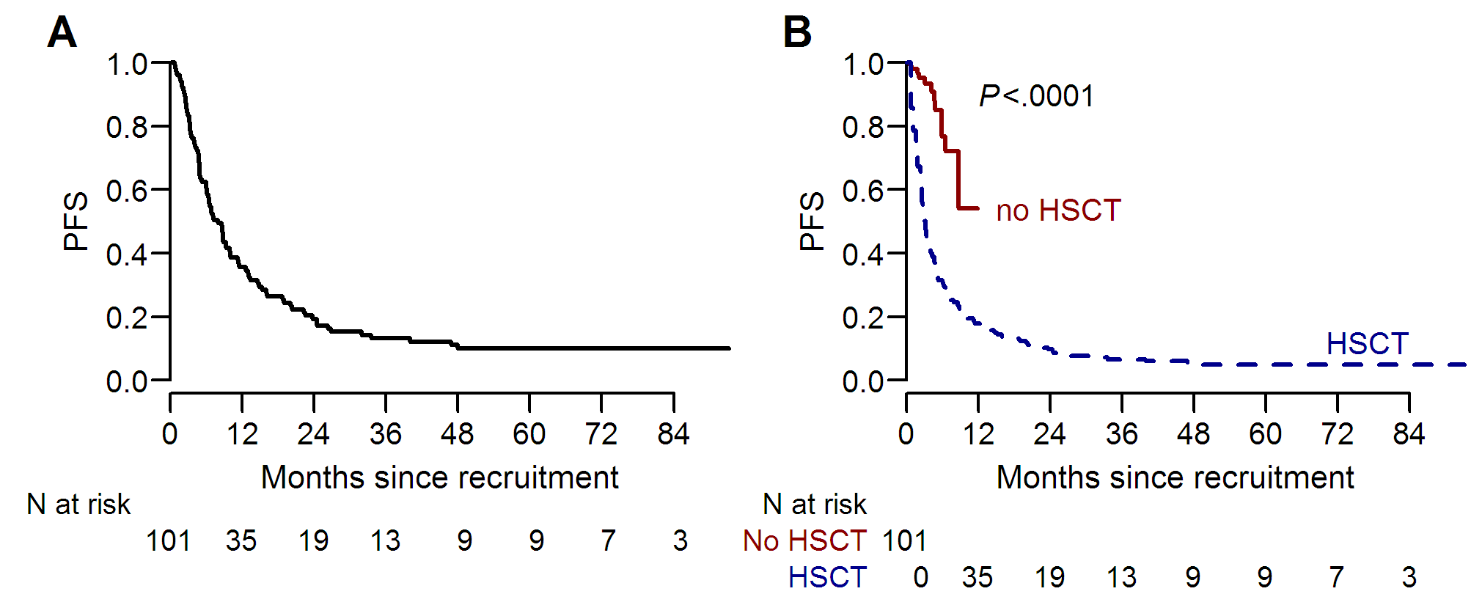


Supplementary Figure 3: Non -relapse mortality (NRM) and cumulative incidence of relapse (CIR) according to disease history (refractory, relapsed)


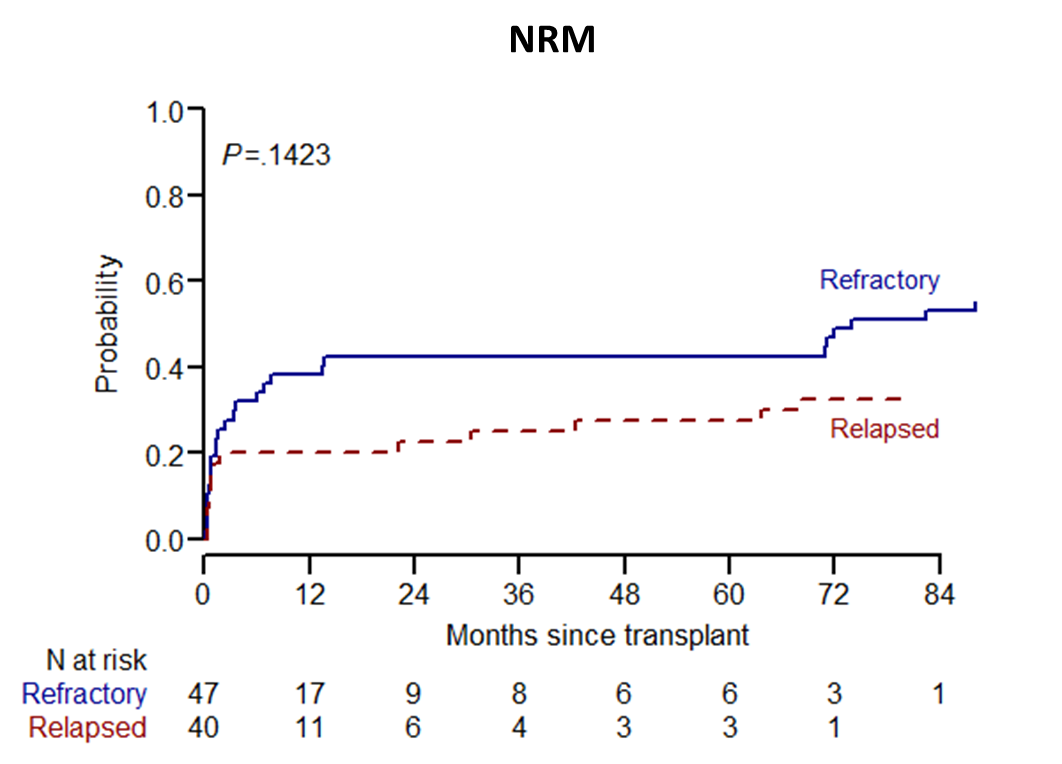

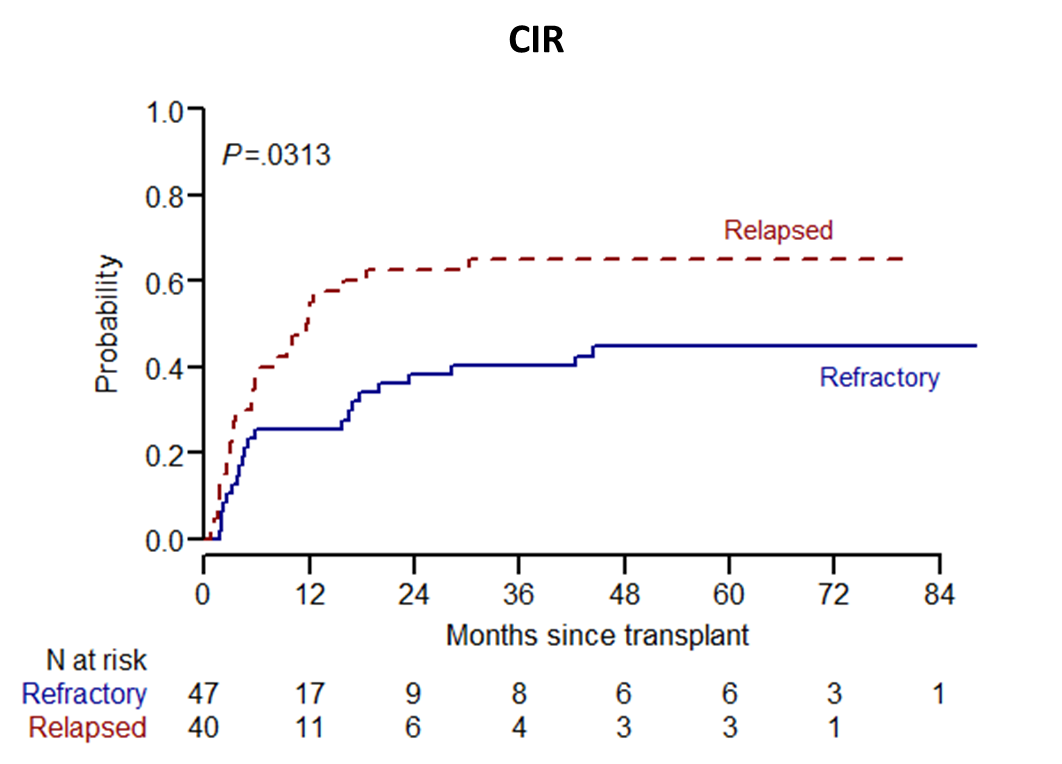

Supplement: Supplementary file 1 — Supplementary material [file 41409_2022_1626_MOESM1_ESM.docx]
